# Developing targeted client communication messages to pregnant women in Bangladesh: a qualitative study

**DOI:** 10.1186/s12889-021-10811-y

**Published:** 2021-04-20

**Authors:** Jesmin Pervin, Bidhan Krishna Sarker, U. Tin Nu, Fatema Khatun, A. M. Quaiyum Rahman, Mahima Venkateswaran, Anisur Rahman, J. Frederik Frøen, Ingrid K. Friberg

**Affiliations:** 1 International Centre for Diarrhoeal Disease Research, Bangladesh (icddr,b), Dhaka, Bangladesh; 2 University of Bergen, Bergen, Norway; 3 Norwegian Institute of Public Health, Oslo, Norway; 4 Tacoma-Pierce County Health Department, Tacoma, WA USA

**Keywords:** Targeted client communication, Antenatal care, Behaviour change technique, Bangladesh

## Abstract

**Background:**

Timely and appropriate evidence-based practices during antenatal care improve maternal and neonatal health. There is a lack of information on how pregnant women and families perceive antenatal care in Bangladesh. The aim of our study was to develop targeted client communication via text messages for increasing antenatal care utilization, as part of an implementation of an electronic registry for maternal and child health.

**Methods:**

Using a phenomenological approach, we conducted this qualitative study from May to June 2017 in two sub-districts of Chandpur district, Bangladesh. We selected study participants by purposive sampling. A total of 24 in-depth interviews were conducted with pregnant women (*n* = 10), lactating women (*n* = 5), husbands (*n* = 5), and mothers-in-law (*n* = 4). The Health Belief Model (HBM) was used to guide the data collection. Thematic analysis was carried out manually according to the HBM constructs. We used behavior change techniques to inform the development of targeted client communication based on the thematic results.

**Results:**

Almost no respondents mentioned antenatal care as a preventive form of care, and only perceived it as necessary if any complications developed during pregnancy. Knowledge of the content of antenatal care (ANC) and pregnancy complications was low. Women reported a variety of reasons for not attending ANC, including the lack of information on the timing of ANC; lack of decision-making power; long-distance to access care; being busy with household chores, and not being satisfied with the treatment by health care providers. Study participants recommended phone calls as their preferred communication strategy when asked to choose between the phone call and text message, but saw text messages as a feasible option. Based on the findings, we developed a library of 43 automatically customizable text messages to increase ANC utilization.

**Conclusions:**

Pregnant women and family members had limited knowledge about antenatal care and pregnancy complications. Effective health information through text messages could increase awareness of antenatal care among the pregnant women in Bangladesh. This study presents an example of designing targeted client communication to increase antenatal care utilization within formal scientific frameworks, including a taxonomy of behavior change techniques.

**Trial registration:**

ISRCTN69491836. Registered on December 06, 2018. Retrospectively registered.

**Supplementary Information:**

The online version contains supplementary material available at 10.1186/s12889-021-10811-y.

## Background

Antenatal care (ANC) provided by skilled health professionals includes risk identification, prevention and management of pregnancy-related or concurrent diseases, health education, and health promotion [1, 2]. Implementation of timely and appropriate evidence-based practices during ANC improves health as well as saves the lives of mothers and newborns [3]. In addition, ANC is a window of opportunity for social, cultural, physiological, and emotional support to pregnant women, through effective communication to increase health care utilization [4, 5]. ANC also provides an opportunity to prevent and manage concurrent diseases through integrated service delivery [6, 7].

Sixty-four percent of pregnant women attend a minimum of four ANC visits worldwide, suggesting the need for context-specific interventions to address ANC utilization [5, 8]. In Bangladesh, 47 % of pregnant women attend four ANC visits, and only 37% of women receive their first ANC before 16 weeks of gestation [9]. This low coverage of ANC may be influenced by socio-cultural beliefs, demographic characteristics, and the performance of the health system [5]. Although physical access has not been reported as a significant problem, utilization is low despite multiple efforts, including demand-side financing projects [10]. Women’s perceptions of care is a key issue limiting service utilization [11, 12]. Women’s negative perceptions of the quality of ANC and the expectation that pregnancy complications are rare may prevent adequate ANC utilization. Therefore, health education about the benefits of ANC is essential prior to, and during, the early stages of pregnancy. It provides an opportunity for dialogue between pregnant women and care providers to promote ANC utilization, birth preparedness, and increase awareness of complications [13, 14].

Maternal knowledge of complications is an essential precondition for routine ANC attendance for identification of early pregnancy-related complications, especially because most of these complications have no symptoms before severe illness develops. Studies from low- and middle-income countries, including Bangladesh, have reported that knowledge about pregnancy complications is low [15–18]. Complications such as anemia, gestational diabetes mellitus (GDM) and hypertensive disorders in pregnancy are common in South East Asia including Bangladesh [19–22]. Therefore, screening for these three conditions during pregnancy is essential, especially given that there are few symptoms at the early stages of these illnesses. Timely ANC attendance is critical for early screening for and identification of anemia, GDM, and gestational hypertension, thereby preventing later complications.

The use of digital health interventions is increasingly prioritized to strengthen healthcare systems, with many low- and middle-income countries implementing digital health technologies to address specific challenges in maternal and child health. Digital health interventions have the potential to improve ANC attendance through behavior change messages and targeted client communication (TCC) [23]. TCC is defined as the transmission of targeted health content to a specified population or an individual within a predefined health or demographic group [24]. In low-income countries, digital health interventions have shown promise as an effective way for TCC to influence behavior and encourage women to access preventive services including ANC [25]. In Bangladesh, about 94% of households have a mobile phone and 60% of currently married women have their own mobile phone [9, 26]. Mobile phone features such as text messaging via Short Message Service (SMS), voice calls, Multimedia Message Service (MMS), or interactive voice recording have been found to be effective in increasing the uptake of different health care services [27–29]. While in some low resource settings, mobile phone text messaging has been shown to increase ANC attendance [30], others have found that telephone call reminders, compared to text messaging reminders, were more effective in increasing ANC attendance [31]. To guide policy-making, more information is needed on which methods can effectively increase ANC utilization in Bangladesh.

The objective of this study was to develop targeted client communication via text messages for promoting antenatal care utilization, as part of an implementation of an electronic registry for maternal and child health. We explored pregnant women’s health-seeking behaviors, knowledge about pregnancy complications, and women’s and families’ perceptions of self-care practices during pregnancy mechanisms that might support the implementation of TCC to increase attendance, and subsequently develop TCC messages guided by these findings. This study is one of the formative components of the eRegMat (electronic registry Matlab) trial, a large cluster-randomized controlled trial to implement and evaluate the effectiveness of an eRegistry on health care services delivered to pregnant women and newborn babies in rural Bangladesh (trial registration number: ISRCTN69491836). In the eRegMat trial, we are comparing an interactive eRegistry (longitudinal client records with health worker decision support and TCC via text messages), developed within the District Health Information System 2 (DHIS2 platform) to a ‘silent registry’ (with longitudinal client records only) on improvements in the quality of care during the antenatal, childbirth, and postnatal periods.

## Methods

### Design

A qualitative research using phenomenological approach, guided by the Health Belief Model (HBM), was carried out to explore perceptions of ANC and pregnancy complications [32]. The HBM focuses on six constructs: perceived susceptibility and severity of complications; perceived benefits of care, self-efficacy; and barriers and cues to action to attend care. We used the HBM to find out individuals’ perceived knowledge of pregnancy care and practice, to predict why and how women seek care during pregnancy. We developed the interview guidelines using HBM constructs for performing in-depth interviews in order to map the lived experience of pregnancy care (see Additional file 1). The interview guidelines focused on anemia, GDM, and hypertensive disorders in pregnancy. Questions on preferred strategies for communication and participants’ views on text message use to address knowledge gaps were also included in the interview guidelines. We pretested all the interview guidelines and based on the feedback, tools were refined and modified for final data collection. The interview guidelines were developed in English and translated to Bangla by the investigators of the study.

### Study site and population

The study was conducted in Matlab South and Matlab North sub-districts in Chandpur District, Bangladesh. The study site mostly consists of rural and riverine delta areas of Bangladesh and is located 80–85 km southeast of Dhaka, the capital of Bangladesh. In Bangladesh, maternal and child health care services are provided by the two separate departments of the Ministry of Health and Family Welfare. Domiciliary community health workers from both departments provide health education on maternal and child health at the village level [33, 34]. At the primary care level, maternal health services are provided at Community Clinics (CC) and Union Health and Family Welfare Centres (UH&FWC) [18]. The UH&FWC cover a population of about 25,000, while CC cover an average of 6000 [33, 34]. The study population included pregnant and postpartum women, husbands, and mothers-in-law of pregnant women. We included husbands and mothers-in-law along with the pregnant and postpartum women as they are the prime household decision-makers of pregnancy-care seeking in this community [35] .

### Sampling

We used purposive sampling to select study participants from the pregnancy register of domiciliary community health workers, based on the inclusion criteria. The following groups were eligible for inclusion in the study: 1) women who were nullipara, primipara or multipara; 2) women with or without antenatal complications during a recent pregnancy; 3) postpartum women who delivered a live baby within 1 month of the interview; 4) husbands of pregnant women; and 5) mothers-in-laws of pregnant women. Husbands and mothers-in-law were not related to the pregnant or postpartum women included in the interviews.

### Data collection

In-depth interviews were chosen as a first step to elicit a vivid picture of the participant’s perspective on and experiences with pregnancy care. In-depth interviews are considered to be a non-threatening method to gather such data. Participants were free to express their true opinions as we assured them that the information shared will not be linked back to the individual participant. Two research investigators with experience in qualitative research conducted all the in-depth interviews. Before the data collection, investigators were well-oriented with the objective of the study as well as the interview guide to foster common understanding of the data collection expectations, processes, and goals. The information collected from the pregnancy register, in terms of inclusion criteria, was verified in the field prior to interviews of the study participants. Research assistants from the study team first contacted eligible participants with a phone call and requested participation in in-person interviews. The two research investigators then visited interested study participants at a time and place convenient and acceptable to them. One female investigator interviewed all the female participants, while the male investigator interviewed all the male participants. We conducted the interviews in the interviewees’ homes or other locations based on their choice. All participants gave informed written consent prior to the interviews and were assigned anonymous study participation numbers before the analysis phase. Each interview took approximately 1 h and was recorded with a digital recorder for transcription. In addition to the in-depth interviews, we also collected background information (see Additional file 2). We conducted the interviews until the data produced little or no new useful information in relation to our study objective. The research team held debriefing sessions at the end of each day of data collection to understand the interpretation of collected data, strengths and weaknesses of interview techniques, and any missed opportunities for further exploration, minimize the confusion among the research investigators, and determine when data saturation was reached. In-depth interviews were conducted from May to June 2017.

### Data analysis

We prepared an outline of the purpose and plan for data analysis. Throughout the data collection process, we listened to the tape-recorded in-depth interviews, and read through all the transcribed interviews to identify themes that were discussed in the interviews. All interviews were conducted in the Bengali language, transcribed, and then translated into English. Data were indexed and coded manually, organized into a matrix, and then sub-themes and themes were generated according to the components of the HBM [36]. Table 1 shows an example of the thematic analysis process. Data were compared between and within different types of respondents to strengthen the appropriateness of the findings. Two researchers in the team with experience in conducting qualitative research individually coded the data and finalized the code list by checking for similarities and dissimilarities during data interpretation. Discrepancies were discussed between the two researchers first, and then with a third researcher until consensus was reached. We analyzed the data to understand the views of the different target audiences on the underlying factors that influence their ANC utilization.

**Table 1:** Thematic data analysis process

Themes	Sub-themes	Codes
Perception of pregnancy complications	• Perceived susceptibility and severity	• Perceived susceptibility to anemia • Perceived susceptibility to diabetes • Perceived susceptibility to hypertension • Perceived severity of anemia • Perceived severity of diabetes • Perceived severity of hypertension • Care seeking • Pregnancy complications
ANC practices	• Perceived benefits of ANC attendance • First ANC attendance • Follow-up ANC visits	• Benefit of ANC • First ANC • Follow-up ANC check-up
Perceived barriers for attending ANC	• Perceived barriers for attending ANC	• Barrier of ANC attendance
Self-care knowledge, practice and family cooperation during pregnancy	• Self-care and family support • Decision-making for maternal care service utilization	• Self -care for pregnancy • Family support • Decision making for care seeking
Targeted client communication strategy	• Client communication strategy • Contact person and time for communication	• Preferred communication channel

*ANC* Antenatal care

### Message development

Using the results from the in-depth interviews based on HBM constructs, we drafted TCC text messages in English that specifically addressed the knowledge gaps and perceptions that contributed to the low uptake of ANC. We tailored these draft messages to be aligned with specific pregnancy conditions, and the timing of screening for these conditions during ANC to increase client acceptance and enhance the impact on service utilization [37].

Once the general topic for each message was agreed upon by the research team, the format of that message was designed based on our review of effective behavior change techniques (BCT) recommended by Abraham and Michie [38]. We applied established theory and behavior change techniques to the development of draft messages to strengthen the potential of achieving specific behavior change [39, 40]. The messages covered six out of 26 BCTs, underpinned by five theories. BCTs included the provision of general information about behavioral risk, information on benefits and consequences of action or inaction of the behavior, encouraging the person to decide to perform, rewarding the effort toward achieving the behavior change, how to perform the desired health behavior, and planning the desired health behavior with a planned outline to perform (Table 2). The theories linked with the six BCTs are the information-motivation-behavioral skills model (IMB), a theory of reasoned action (TRA), theory of planned behavior (TPB), social-cognitive theory (SCogT) and control theory (CT). We matched each component of a given draft message to a specific BCT. We used positively framed messages as opposed to negatively framed messages to engage in healthy behavior [41]. To address the knowledge gaps identified through the HBM constructs, we included information on common pregnancy complications of anemia, hypertension, and diabetes, their consequences, and the benefits of ANC attendance. To nudge the study participants’ decisions towards healthy behavior, messages were personalized using their name [42, 43]. The content of these draft text messages was translated into the Bengali by the research team.

**Table 2:** Behavior change techniques and HBM used to create TCC messages for ANC utilization

Behavior change technique [38]	Description	HBM domain	Example text message phrase
Provide information about behavior health link (IMB)	General information about health outcomes concerning behavior	Perceived benefit	Come to our health center, and we will check you and your baby (ies’)’s health throughout your pregnancy.
Provide information on consequences (TRA, TPB, IMB, SCogT)	Information about the advantages and disadvantages of action	Perceived susceptibility and severity	High blood pressure may develop during this time and lead to a serious problem for the mother. The baby might be born early or very small.
Prompt intention formation (IMB, SCogT)	Encourage the person to decide to act	Other	Come to our health center, and we will check your and your baby’s health throughout your pregnancy.
Provide general encouragement (SCogT)	Outcome of performance	Perceived benefits	Anemia will be treated accordingly, and you will be better prepared for delivery.
Prompt specific goal setting (CT)	Involves detailed planning of ANC visits	Cues to action	Please remember to attend your ANC visit next week. Excess blood pressure in pregnancy may cause serious problems for the mother and the baby. Come to the health centre, and we will reassess your blood pressure.
Provide instruction (SCogT)	Tell the person how to perform the desired behavior and how to prepare for it.	Cues to action	Please remember to attend your ANC visit next week. Come to our health center.
Personalization (nudge theory)	Insertion of the recipient’s name and signing with the name of the clinic as a “trusted source”	Other	Salam (name) apa. Name of health facility

*IMB* Information-motivation-behavioral skills model, *TRA* Theory of reasoned action, *TPA* Theory of planned behaviour, *SCogT* Social –cognitive theory, *CT* Control Theory

Two focus group discussions were conducted with pregnant women to get their feedback on the draft messages to ensure that the messages were easily understandable and acceptable. Specifically, participants were shown the content of the messages and asked to comment on their understanding of the message and how to improve the specific text and language. Women were selected from community health workers’ pregnancy registers. The results from the focus group discussions were evaluated by the team and the final Bengali text messages were modified based on the results.

### Ethical considerations

This study was approved by the ethics and research committees at the International Center for Diarrheal Disease Research, Bangladesh (protocol number: 16054), and exempt from review by the Regional Ethical Committee in Norway, Southeast region (2017/1018/REK sør-øst C). Individual informed written consent was obtained from all participants.

## Results

### Characteristics of the study participants

Of a total of 24 in-depth interviewees (Table 3), ten were pregnant women, five were postpartum women, four were mothers-in-law, and five were husbands. All pregnant women were housewives from 18 to 26 years of age. Among the ten pregnant women, four were pregnant for the first time. At the time of the interview, the mean gestational age was 23 weeks and 4 days (8–36 weeks). Seven pregnant women had less than 10 years of education, and the rest had more than 10 years. All the postpartum women were housewives, from 22 to 32 years of age. Three postpartum women had completed less than 10 years of education and two women, more than 10 years. The five husbands ranged from 25 to 41 years of age; four had less than 5 years of formal education, and one up to 10 years.

**Table 3:** Characteristics of study participants

Participants	Mean age in years (range)	Education level (years)	Household type	Parity
Pregnant women	22 (18–26)	Secondary (6–10): 7 Higher secondary (> 10):3	Single family: 3 Extended family: 7	Nullipara: 4 Multipara: 6
Postpartum women	25 (18–32)	Secondary (6–10): 3 Higher secondary (> 10):2	Single family: 2 Extended family: 3	Primipara:1 Multipara: 4
Mothers-in-law	55 (50–60)	Primary (0–5): 3 Secondary (6–10): 1		
Husbands	35 (28–41)	Primary (0–5): 4 Secondary (6–10): 1		

### Perception of pregnancy complications

#### Perceived susceptibility and severity

Pregnant and postpartum women mentioned some pregnancy complications from their personal experiences. Many of them got information from health care providers. Besides, they also learned from observing the experience of their family members, and others in their neighborhood. In this context, one pregnant woman said, “*I have heard that many women face problems. Some suffer a lot during delivery. A girl from our neighborhood delivered her baby at the eighth month, she also suffered from high blood pressure.*” (Pregnant woman, para 0).

Women with children mentioned more pregnancy complications compared to women who had never given birth. Complications reported by pregnant and postpartum women included excess vomiting, less fetal movement, anemia, breech presentation, high blood pressure, convulsion, “white discharge”, and fever. Most mothers-in-law mentioned that headache, abdominal pain, vaginal bleeding, high blood pressure, jaundice, tuberculosis, and pneumonia could develop during pregnancy. The majority of the husbands reported that very few complications could happen in pregnancy, such as vomiting, reduced food intake, abdominal cramp or pain, and edema. One husband stated that “*this is a matter of women, and* *we do not understand the female health (pregnancy-related) problem.*” (Husband, CNG driver).

Women were asked to discuss common complications related to pregnancy, and then we focused on three specific complications: anemia, hypertension, and GDM. Most of the postpartum women spontaneously mentioned high blood pressure as a pregnancy complication and convulsion as a consequence. One postpartum woman said, “*If the pressure rises, that could create problems. If it’s high, some may have convulsions during pregnancy. I saw someone who had convulsions due to pressure. Then again, low pressure is also bad.”* (Postpartum woman, para 4).

On the other hand, only a few pregnant women spontaneously mentioned high blood pressure and convulsion. Most of the husbands and mothers-in-law did not mention high blood pressure and its consequences.

Anemia was rarely mentioned as a perceived pregnancy-related complication by pregnant and postpartum women. One pregnant woman stated after probing, *“I have heard that anemia could happen during pregnancy. But, I can’t say what can happen to the mother and baby due to anemia. I have no complete idea.”* (Pregnant woman, para 1). Few of the pregnant and postpartum women linked anemia only to blood loss during delivery. They thought that women were less likely to develop anemia at their first pregnancy. Women with previous births could develop anemia in the current pregnancy due to blood loss at their previous delivery. Related to this, one pregnant woman stated, *“This is my first pregnancy; I should have plenty of blood in my body*.” (Pregnant woman, para 0).

Few husbands stated anemia and weakness as a consequence, while mothers-in-law could not report on anemia at all. Most of the respondents did not mention that diabetes could develop during pregnancy. One postpartum woman stated, “*Many pregnant women develop diabetes after delivery … my elder brother’s wife did not have any disease previously, but after delivery of her last child, diabetes was diagnosed.”* (Postpartum woman, para 2).

#### Care for complications

When we asked participants what they would do for any pregnancy-related complications, all stated that they would visit a qualified doctor if they perceived it as an emergency. One postpartum woman stated that pregnant women with anemia should take iron tablets and eat nutritious food. One pregnant woman said that “*If I suffer from any problem, I should visit a doctor and (I) need to follow doctor’s advice*.” (Pregnant woman, para 2).

### Antenatal care practices

#### The perceived benefit of ANC attendance

Almost all pregnant and postpartum women mentioned that ANC is necessary to know the status of the baby and mother but could not mention the importance of timely ANC as preventive care for appropriate screening and management. ANC was mostly viewed as necessary during pregnancy only if complications developed. In addition, women also thought that the health care provider needs to inform pregnant women about ANC utilization.

One pregnant woman with two children and in her fifth month of pregnancy stated, “*I am well now, it is not necessary to go to the healthcare provider now, and they (health care provider) haven’t even called me yet … if any healthcare provider calls me for ANC, I will only go for an ANC visit, but if she (health care provider) doesn’t come to me, I will not go for it. It is her (health care provider’s) duty to inform me to take ANC, not mine.*” (Pregnant woman, para 2).

Another postpartum woman stated the benefits of ANC, “*The benefits of check-ups are that I could get to know the position of the baby, whether the baby is in the upper or lower abdomen. I could also get to know whether the baby is healthy or not.*” (Postpartum woman, para 2).

Mothers-in-law also stated that ANC is needed to know the status of the baby and the mother but could not mention the importance of timely ANC for appropriate screening and management of complications. Similar knowledge was found among husbands, although one of them stated that ANC is important to see the growth of the baby, and to identify problems. Another husband stated that there is no need for ANC during pregnancy. He mentioned, “*I don’t think that ANC is important, as my wife did not suffer from any problem, I did not give any importance to ANC. If she suffered from any complication, I would have gone to the doctor with my wife and ask (ed) about the problem*” (Husband, cook).

#### First ANC attendance

The perceived need to attend ANC within the first 5 months of pregnancy was very low among the pregnant women. One pregnant woman at her 4 months of gestational age quoted, “*I have a plan to visit the facility for my check-up (ANC) between five to six months (of gestation).*” (Pregnant woman, para 0).

Another pregnant woman at the same gestational age said, “*No, I don’t need to visit the health facility yet as I am now four months pregnant.*” (Pregnant woman, para 1).

Few pregnant women talked about the high chance of miscarriage as the reason behind not seeking ANC at an early stage of pregnancy. The chance of miscarriage within 5 months was believed to be high, and the women thought that ANC does not play any role in preventing miscarriages.

On the other hand, most of the postpartum women had received ANC, and the majority had attended ANC following complications during their pregnancy or f during a previous pregnancy or delivery.

#### Follow-up ANC visits

Most of the women did not receive information on the timing of follow-up ANC visits from health care providers and did not attend follow-up ANC visits. A few women received follow-up ANC visits, but it was not timely as per the ANC visit schedule. The women reported attending follow-up visits when they faced complications, and in this regard, one pregnant woman said, “*I visited a doctor for excess vomiting, and she (doctor) prescribed medicine. But the condition is the same, although I am taking medicine. I am at six months now. Still, I am vomiting. During the first visit, I was told that it would subside gradually. If it happens more frequently, then I have a plan to visit a doctor.”* (Pregnant woman, para 2).

Similarly, most of the husbands did not know about the necessity of follow-up ANC visits. Few of them learned about the needs of ANC visits after they became fathers. In this regard, one husband narrated “*I have a child, if I did not have a child, I would not have known about the check-ups. After three months and up to delivery, we need to go monthly for check-ups.”* (Husband, CNG driver).

### Perceived barriers to ANC attendance

Pregnant women who did not attend care reported a wide range of reasons, including lack of decision-making power, distance to the facility, being too busy, not being satisfied with the treatment by health care providers, non-cooperation by their husband, and unavailability of any family member to help in the household. Regarding the non-cooperation by a husband, one woman quoted, “*I had bleeding yesterday, and I cried a lot. I knew a little bit about what I should do for this. But I was unable to do anything (she was pointing to her husband through a non-verbal expression that her husband did not take her to the doctor while she was bleeding). If I can’t go to the doctor now, if I continue bleeding, it would be harmful to my baby and for me as well. But I cannot do anything.*” (Pregnant woman, para 2).

### Self-care knowledge, practice, and family cooperation during pregnancy

#### Self-care and family support

We further explored women’s general attitudes to taking care of their pregnancy through self-care and their families’ supportiveness of her pregnancy and care. The majority of women practiced healthy behaviors such as eating adequate nutritious diets, avoiding heavy work, and resting. Most mothers-in-law and husbands knew that pregnant women need to eat nutritious food, take proper rest, and avoid lifting heavy things. Very few first-time pregnant women mentioned dietary restrictions such as avoiding selected fish and green coconut during pregnancy.

Most of the women living with extended families received household support from their family members, especially from mothers-in-law. “*My mother-in-law brings water from outside, sweeps the room, and helps me to cut the vegetables. I do all the cooking and other work.*” (Pregnant woman, para 2). Women living in single-family households generally did not get support for their household work. One pregnant woman living in a single-family household reported, *“I am the only female in the household. I have two small children. I need to do some heavy work, I bring water for cooking and washing clothes myself.*” (Pregnant woman, para 2).

### Decision making for maternal care service utilization

When the pregnant and postpartum women were asked about the decision-maker for their health care service utilization, most of them mentioned that their husbands were the main decision-makers in the family. But nulliparous women reported that their mothers-in-law played a vital role in the decision making along with their husbands. One pregnant woman stated, “*I became pregnant for the first time. I don’t know much about pregnancy. So, most of the decisions are taken by my mother-in-law and husband.”* (Pregnant woman, para 1).

All mothers-in-law with currently pregnant daughters-in-law mentioned that she and her son jointly made critical decisions about the pregnancy-related care in the family. On the other hand, most husbands said that they were the main decision-maker in the family. In this context, one husband cited, “*I will have to make the decision. I cannot depend on anybody. Now I am here; I will make the decision.*” (Husband, migrant worker).

### Targeted client communication strategy

#### Modes of contact and reminders

Pregnant women were asked about communication strategies that could increase ANC attendance. Almost everyone preferred direct phone calls to remind them of ANC dates and give them health information. In favor of phone calls, one woman stated, “*Through a phone call, I would be able to talk directly. In the case of messages, I would have to read the message to understand. Sometimes I read (the) message, sometimes not, if I am not busy with any work. That’s why phone calls would be good*.” (Pregnant woman, primipara)*.* Another woman stated, “*If there is an emergency and the phone rings, I would pick it up and listen to that. After that, the phone remains somewhere idle; the message would only make a small sound, nothing after that*.” (Postpartum woman, para 3). However*,* most of the participants mentioned that text messages could work as a reminder for the ANC schedule.

#### Contact person and time for communication

Given that husbands and mothers-in-law were the decision-makers, we asked to whom and when to send the text messages related to ANC attendance and pregnancy complications. Most women owned a mobile phone and suggested that they preferred direct communication with the pregnant woman. Very few women did not own mobile phones and suggested communicating with their husbands and mother-in-laws. Although some said the text messages could be sent at any time, others suggested that evenings would be better.

### Development of SMS messages for TCC

The following main findings from our study guided the development of SMS messages for TCC. First, women and families only perceive the need for early ANC when women are sick. Perceived susceptibility to common pregnancy complications, and the knowledge that they may occur without symptoms, was low among study participants. Second, women did not recognize that common pregnancy complications could necessitate care during pregnancy. Third, they did not know that attending ANC could result in fewer complications or earlier detection of complications. Fourth, they were unaware of the appropriate number and timing of ANC visits. Finally, most of the women who received detailed information on when and where to attend follow-up ANC during their first ANC visit did attend those follow-up visits, suggesting that lack of information is a key factor leading to inadequate utilization of ANC and that basic information served as cues-to-action. The evidence did not suggest any specific modifiable factor which could address a majority of women’s self-efficacy and ability to attend ANC.

Given this evidence, and the planned integration of automated data-driven SMS messages into the eRegistry, we designed a series of text messages for women to act as cues-to-action and target women’s knowledge about ANC, specifically on 1) the benefits of attending ANC; 2) women’s susceptibility to complications; and 3) the severity of complications. The messages were designed to match the timing of critical screenings for common conditions such as anemia, hypertension, and GDM. We used clinical data from the eRegistry to further tailor these messages, based on women’s gestational age and common risk factors for the medical conditions targeted (Table 4), in order to make the text messages more individualized and provide added value to the individual woman.

**Table 4:** Common Risk Factors for Anemia, Hypertension, and GDM

Risk factors for anemia	Risk factors for hypertension	Risk factors for GDM
Age < 20	Age ≥ 35 [44]	Age > 25 [45]
Low Body Mass Index [46, 47]	High body weight/high Body Mass Index [44]	High Body Mass Index [45, 48]
Grand multiparity [49, 50]	Nulliparity [20, 51]	Grand multiparity [52]
Multiple pregnancies [50]	Previous hypertension [51]	Multiple pregnancies [45]
	Previous Small for Gestational Age [53, 54]	Previous hypertension [45]
	Previous prenatal mortality [55, 56]	Previous GDM [45]
	Family history of hypertension [44, 51]	Previous perinatal mortality [55]
		Family history of DM [45]

*GDM* Gestational Diabetes Mellitus, *DM* Diabetes Mellitus

The Bangladeshi government recommends a schedule of four ANC visits for low-risk pregnant women. Reminder messages were designed to be sent 1 week and 1 day prior to the scheduled ANC date; we selected one topic to be highlighted in each of the reminder messages (Table 5). In addition, we created a welcome message to be sent upon enrolment, referral facilitation messages, and facility delivery reminders. The referral facilitation messages were reminders to women that their provider had recorded danger signs or created a referral in the eRegistry.

**Table 5:** National ANC schedule and linked health topic for messages as recommended by the national guideline

ANC schedule	Health topic for messages
Within 16 weeks	Anemia [7, 57, 58]
24–28 weeks	Gestational Diabetes Mellitus [59, 60]
32 weeks	Gestational Hypertension [57, 58]
36 weeks	Malpresentation [57, 58]

Given that the messages would be automatically generated within the eRegistry, we were able to additionally tailor each message based on clinical characteristics. Specifically, if data were entered into the eRegistry indicating anemia, hypertension, or gestational diabetes, an algorithm was developed which would take that into consideration and appropriately modify the messages. And, if the risk factors for these conditions were documented in the eRegistry (as shown in Table 4), the text messages would be tailored further.

The initial draft of the TCC messages, based on the HBM and the ANC schedule, and tailored with the behavior change techniques, were translated into Bengali from English and shared with pregnant women in focus group discussions (data not shown). Participants reported that the content of messages was adequate, but some modifications of terminology were needed for ease of understanding. We then reviewed each message and finalized the wording. Each message was adapted to the typical SMS character limits (knowing that for some messages up to three SMS could be sent). In the end, a library of 43 TCC messages was developed based on whether the individual woman had anemia, hypertension, GDM, and associated risk factors in the eRegistry. Examples of the messages are included in Table 6.

**Table 6:** Selected examples of the final targeted TCC messages

Message type	Message Timing	Message Content
Welcome message	same day as pregnancy identification/enrolment	Welcome [xxx] apa. We will provide four routine ANC visits. Come to our health centre and we will check you and your baby (ies’)’s health throughout your pregnancy. [yyy]
Visit 1	1 week prior to the first scheduled ANC visit	Salam [xxx] apa. Please remember to attend your ANC visit next week. 1 in 3 women is anemic in pregnancy. Anemia can cause dizziness and weakness. Come to our health centre and we will check for your anemia and will treat accordingly so that you will be better prepared for delivery. [yyy]
Visit 2 reminder - for women with no risk	1 week prior to second scheduled ANC visit	Salam [xxx] apa. Please remember to attend your ANC visit next week. We will check for sugar in blood or urine for signs of diabetes. Diabetes in pregnancy has to be followed-up carefully throughout pregnancy to ensure that both mother and baby stay healthy. [yyy]
Visit 2 reminder – for women with mild or moderate anemia	1 week prior to second scheduled ANC visit	Salam [xxx] apa. Please remember to attend your ANC visit next week. Anemia in pregnancy may cause problems for the mother and the baby, if not treated. Come to the health centre and we will reassess for anemia and check for diabetes. [yyy]
Visit 3 reminder - for women with no risk	1 week prior to third scheduled ANC visit	Salam [xxx] apa. Please remember to come to ANC this week as agreed. High blood pressure may develop during this time and lead to serious problems for the mother. The baby might be born early or very small. Come to ANC and we will measure your blood pressure and manage it appropriately. [yyy]
Visit 3 reminder - for women with severe hypertension	1 week prior to third scheduled ANC visit	Salam [xxx] apa. Please remember to attend your ANC visit next week. Excess blood pressure in pregnancy may cause serious problems for the mother and the baby. Come to the health centre, and we will reassess your blood pressure. [yyy]
Visit 4 reminder - for women with no risk	1 week prior to fourth scheduled ANC visit	Salam [xxx] apa. Please remember to attend you ANC visit this week. Knowing your baby’s presentation at this time can help you plan a safe delivery. Come to the health centre and we will check your baby’s position. [yyy]
Visit 4 reminder - for women with anemia and GDM	1 week prior to fourth scheduled ANC visit	Salam [xxx] apa. Please remember to attend your ANC visit next week. Knowing the baby’s current position helps you to plan for safe delivery. Come to the health centre, and we will check your baby’s position and reassess your diabetes and anemia. [yyy]
1-day reminder	1 day before each routinely scheduled ANC visit	Salam [xxx] apa. Remember to attend your ANC visit tomorrow. It is very important to know your present health status. [yyy]
Facility Delivery	1 week after last 36-week message	Salam [xxx] apa. Your health history puts you at greater risk of problems. You are advised to give birth in a facility for a safer delivery. [yyy]
Danger Sign	1 day after being diagnosed	Salam [xxx] apa. You were referred to a higher level facility for improved care yesterday. Please attend care immediately if you have not already done so. [yyy]

[xxx]: woman’s name; [yyy]: name of health facility; *GDM* gestational diabetes mellitus

## Discussion

The study found that most of the participants could not describe ANC as a preventive form of care. It was viewed as necessary during pregnancy only if complications developed, while the WHO recommends preventive ANC for early identification of complications [5]. We found that most women would only attend ANC after 5 months of pregnancy due to concerns about miscarriages. They would seek care regardless of their gestational age if complications arise. In general, the population had limited knowledge of ANC, pregnancy complications, and the consequences for the mothers and their neonates. Limited ANC attendance has been shown to result in poor pregnancy outcomes [61–63]. The utilization of ANC was low, and women did not receive ANC services at the recommended time. Similar to other reports from Bangladesh, only a small proportion of women in our study received their first ANC visit within 16 weeks of gestation [9]. Most pregnant and postpartum women mentioned that ANC is required only to know the wellbeing of the baby and the mother. Women also reported not having adequate information on the timing and number of ANC visits needed in our study. A cross-sectional study found similar results and observed low levels of awareness about ANC among pregnant women in a rural area, and recommended development and strengthening of behavior change communication [64].

Poor understanding of complications, combined with the lack of contextual health education, might lead to less timely attendance at ANC. Similar to our findings, another study in Matlab, Bangladesh, reported that only 26% of women had good knowledge of pregnancy complications [18]. Several studies have shown that ANC utilization is associated with women’s knowledge about pregnancy complications [18, 65, 66]. Women with poor knowledge of pregnancy complications are also likely to be unfamiliar with the consequences of the conditions. Awareness of the benefits of ANC is positively associated with maternal and neonatal outcomes [67]. However, we know that knowledge alone without adequate access, affordable costs, and community support will not improve utilization.

Lack of decision-making power of women for using maternal and child health care may also contribute to low ANC utilization in Bangladesh. Women need to be empowered with adequate health information, availability, and use of services to make their own decisions regarding healthcare through different strategies [68, 69]. Although husbands and mothers-in-law are the primary decision-makers in the household and make the majority of decisions about health service utilization during pregnancy and delivery, researchers perceived that their knowledge of pregnancy complications was limited. Creating awareness among husbands and mothers-in-law using health education in simple language can potentially improve maternal and neonatal service utilization [70]. Although health information is essential for women’s empowerment, adequate support in terms of availability of and access to services and additional structural factors relating to household decision-makers are needed in addition to achieve the goal.

The prevalence of mild to moderate anemia during pregnancy is about 50% in rural Bangladesh, and early diagnosis and management can prevent serious consequences for the health of the mother and the baby [71]. The inadequate knowledge of anemia during pregnancy demonstrated by the participants in our study is similar to the other studies conducted in low-and-middle-income countries [72, 73]. Lack of knowledge on anemia could be one reason for the high prevalence of anemia in Bangladesh. Our finding of poor knowledge of GDM is aligned with studies conducted in Bangladesh and other low-and-middle-income countries [74, 75]. Compared to diabetes and anemia, knowledge of hypertension was better, especially among postpartum women, presumably because some had developed hypertension during their pregnancy. A study conducted in Bangladesh assessing community awareness, beliefs, and experiences around hypertension found a lack of knowledge of hypertension [76]. This low level of knowledge about anemia, hypertension, and diabetes implies that effective health education could improve adequate knowledge, thereby increasing ANC utilization among pregnant women.

Although studies have reported that text message interventions can increase ANC utilization [25], knowledge of women’s reading skills, access to mobile phones, and willingness to receive SMS are all essential to appropriately tailor these messages and increase uptake of ANC. Our study found that most pregnant and postpartum women owned mobile phones and were educated beyond the primary level. Though women expressed their willingness to receive SMS in our study, their age and current practice of mobile phone usage might ultimately influence ANC utilization.

The study has some limitations. Perceived knowledge of ANC care might be different in other parts of Bangladesh due to differences in context. Another weakness might be the challenges that the study participants might have faced in putting their experiences, meanings, and emotions into words. Qualitative methods alone may not be adequate for understanding the magnitude of the problem. We could not explore other factors that might influence their knowledge and practices, such as participants’ exposure to mass media and their social capital. We did not cover a wider range of participants such as health care providers, health managers, local elite, and policy makers that might have contributed to more comprehensive insight.

To our knowledge, this is the first qualitative study in Bangladesh aiming to develop TCC via text messages based on community perceptions for the purpose of increasing ANC utilization. The study shows the critical need to evaluate the local beliefs and belief structures when developing any form of client communication, especially when it will be delivered through an intermediary, such as a digital device. Key strengths of the study design and the development of TCC include having the researchers involved in all steps towards the final cluster-randomized trial of the comprehensive eRegistry, to ensure that the messages are well-integrated within the larger health systems platform. To enhance the appropriateness of the data analysis, we checked transcripts against audio recordings, and performed triangulation among codes and themes by consensus. We placed special emphasis on confidentiality, particularly important in this context where we were linking clinical records with text messages that could be read by anyone that has access to the phone. As an example, recipients’ specific risk factors were excluded from the text messages, considering that a woman might share her mobile phone with other family members. With due consideration to the cultural context, we decided to send the TCC messages 1 week prior to scheduled visits and a second reminder message 24 h before the visit to empower women for future planning and to react quickly if necessary. The output of this study is a critical component to the eRegMat trial, which is, in part, aimed at assessing the effectiveness of these automated TCC messages to increase ANC utilization. This study’s findings are relevant for any implementations of digital health interventions to increase ANC coverage and can serve as a model for a theoretically informed approach to TCC.

## Conclusion

This study presents an example of TCC message development based on systematically evaluated gaps in knowledge, awareness of pregnancy care and complications, established theory, and behavior change techniques with the purpose of increasing ANC utilization. Improving knowledge of pregnancy complications and the importance of timely ANC is essential, and providing health information through text messages has the potential to raise awareness among pregnant women. A text message intervention is feasible in this Bangladeshi population as most women and households have access to mobile phones. The effectiveness of these TCC messages is being evaluated in an ongoing cluster-randomized controlled eRegMat trial (trial registration no: ISRCTN69491836).

## Supplementary Information

**Additional file 1.** In-depth interview guides.

**Additional file 2.** Background information.

## Data Availability

The datasets and materials used in the study are available from the corresponding author on reasonable request.

## Abbreviations

ANC: Antenatal care
GDM: Gestational diabetes mellitus
TCC: Targeted client communication
SMS: Short Message Service
MMS: Multimedia Message Service
eRegMat: Electronic registry Matlab
DHIS2: District Health Information System 2
HBM: Health Belief Model
CC: Community Clinic
UH&FWC: Union Health and Family Welfare Centres
DM: Diabetes Mellitus
BCT: Behavior change technique
IMB: Information-motivation-behavioral skills model
TRA: Theory of reasoned action
TPB: Theory of planned behavior
SCogT: Social-cognitive theory
CT: Control theory
