# Supplementary material for: Developing targeted client communication messages to pregnant women in Bangladesh: a qualitative study
Source: BMC Public Health. 2021 Apr 20;21:759. doi: 10.1186/s12889-021-10811-y (PMC8056650; doi:10.1186/s12889-021-10811-y)
Supplement: Supplementary file 1 — Additional file 1. In-depth interview guides. [file 12889_2021_10811_MOESM1_ESM.docx]

# In-depth interview guides

All of the in-depth interviews will be guided to elicit answers relating to the health belief model. That is the perceived susceptibility and severity of health complications during pregnancy, as well as the barriers and benefits, self-efficacy, and cues to action to attend antenatal care.

The interview guides include anemia, hypertension, and diabetes, but during the interviews, local terms will be used as these terms are well known in the general public.

# In-depth interview guide for Pregnant women

**Perception of pregnant women about Pregnancy Care**

How do you take care of yourself during pregnancy? Do you think that a woman needs some extra care during her pregnancy? If you think so, what kind of care are required during pregnancy? From whom you got information about care during pregnancy?

What should a woman do or should not do during her pregnancy? [use hints: restriction to go outside, not to do work, food intake, etc.]

Who are the family members live with you? What kind of support do they provide during your recent pregnancy (ask separately for each adult family members even including young children as they sometimes help in HH chores)

How are decisions taken about your care? [About staying in natal /in-laws home, having food, antenatal care, etc.]

If you want to go to the facility for antenatal care, how you inform your family members? How your family member can help you to attend the visit? Do family members taking care of you follow the advice of Health care provider?

What complications/danger signs can occur during pregnancy? Source of information, compliance/non-compliance with the danger signs.

What will happen if pregnant women suffer from these danger signs? If pregnant women have any complications, what should the women and their families do? What can happen if they do not treat the complications?

Have you heard about anemia (pallor)/ hypertension (pressure)/ diabetes mellitus (sugar in the blood) like health problems during pregnancy?

How can a person identify these health problems during pregnancy?

If women suffer from anemia (pallor), hypertension (pressure), and Diabetes mellitus (sugar in the blood), what should do the pregnant women and her family members? What are the things (you and your baby) can happen if women do not treat these health problems? How can the complications be prevented for keeping pregnant women and her child healthy during pregnancy?

Do family members have any role in taking care of pregnant women during pregnancy with complications? Do health care providers have any role in taking care of women during pregnancy with complications?

**Awareness and perception regarding ANC, source of information, issues of ANC, Places to go for ANC**

What do you know about routine antenatal care visits during pregnancy? Do you think it is necessary at all for a woman during her pregnancy? If yes, why and when? If no, why not?

*If the woman did attend ANC visits:* Have you received any antenatal care visits? If so, from where and whom? How frequently or how many times you have to go for ANC visit? Were you asked to attend a follow-up visit? Could you attend all the follow-up visit? If you missed any follow-up visit, then what did you do? How could you remember about your follow up visit date? What benefit did you get from ANC?

What problems did you face to attend any of your ANC visits? (Hints: could not remember, cost, distance, unavailability of providers, the family did not support, did not have any complication, etc.)

What advice given to you during ANC? Did you get any information from your ANC provider regarding danger signs/complications during pregnancy?

*If the woman did not receive ANC visit:* why did not you receive any ANC?

**ANC reminder and messages**

How can we effectively remind pregnant women about follow up ANC visits?

Will it help you if you receive a reminder for ANC visit through SMS to your mobile phone? What type of reminder would you like to get? What would be easier for you to understand the reminder and remember it? (Hints: SMS in Bengali or English, Voice call, voice SMS, etc.)

How can we aware of a pregnant woman and her family member if she found any risk factor during her ANC check-up so that she can visit qualified health providers at the right time? [Hints: SMS in Bengali or English, Voice call, voice SMS, etc.].

To whom should we contact for sending messages directly to a pregnant woman or her family members or both at a time?

Any questions you want to ask? Any suggestions you want to put forward?

*Thank you for your time and participation! We value all your comments and opinions. Together we will develop effective ways of improving the quality of antenatal care.*

# In-depth interview guide for postpartum women

**Perception of postpartum women about Pregnancy care**

When, where, and by whom you delivered the baby?

How did you take care of yourself during your last pregnancy? Do you think that a woman needs some extra care during her pregnancy? If you think so, what kinds of care are required during pregnancy? From whom you got information about care during pregnancy?

What should a woman do or should not do during her pregnancy? [use hints: restriction to go outside, not to do work, food intake, etc.]

Who are the family members live with you? What kind of support did they provide during your last pregnancy (ask separately for each adult family members even including young children as they sometimes help in HH chores)

How were decisions taken about your care? [About staying in natal /in-laws home, having food, antenatal care, etc.]

When you planned a visit to the facility for antenatal care, how you inform your family members? How did your family member help you to attend antenatal care visits? Did you follow advice on what was given by the provider? How your family members took care of you to follow the advice?

What complications/danger signs can occur during pregnancy? Source of information, compliance/non-compliance with the danger signs. Did you face any complications during your pregnancy? If yes, how was the complication identified? For complications, where did you go?

What will happen if pregnant women suffer from these danger signs? If pregnant women have any complications, what should the women and their families do? What can happen if they do not treat the complications?

Have you heard about anemia (pallor)/ hypertension (pressure)/ diabetes mellitus (sugar in the blood) like health problems during pregnancy?

How can a person identify these health problems during pregnancy?

If women suffer from anemia (pallor), hypertension (pressure), and Diabetes mellitus (sugar in the blood), what should do the pregnant women and her family members? What are the things (you and your baby) can happen if women do not treat these health problems? How can the complications be prevented for keeping pregnant women and her child healthy during pregnancy?

Do family members have any role in taking care of pregnant women during pregnancy with complications? Do health care providers have any role in taking care of women during pregnancy with complications?

**Awareness and perception regarding ANC, source of information, issues of ANC, Places to go for ANC**

What do you know about routine antenatal care visits during pregnancy? Do you think it is necessary at all for a woman during her pregnancy? If yes, why and when? If no, why not?

*If the woman did attend ANC visits:* Have you received any antenatal care visits? If so, from where and whom? How frequently or how many times you have to go for ANC visit? Were you asked to attend a follow-up visit? Could you attend all the follow-up visit? If you missed any follow-up visit, then what did you do? How could you remember about your follow up visit? What benefit did you get from ANC?

What problems did you face to attend any of your ANC visits? (Hints: could not remember, cost, distance, unavailability of providers, the family did not support, did not have any complication, etc.)

What advice given to you during ANC? Did you get any information from your ANC provider regarding danger signs/complications during pregnancy?

*If the woman did not receive ANC visit:* why did not you receive any ANC?

**ANC reminder and message**

How can we effectively remind pregnant women about follow up ANC visits?

Will it help you if you receive a reminder for ANC visit through SMS to your mobile phone? What type of reminder would you like to get? What would be easier for you to understand the reminder and remember it? (Hints: SMS in Bengali or English, Voice call, voice SMS, etc.)

How can we aware of a pregnant woman and her family member if she found any risk factor during her ANC check-up so that she can visit qualified health providers at the right time? [Hints: SMS in Bengali or English, Voice call, voice SMS, etc.].

To whom should we contact for sending messages directly to pregnant women or her family members or both at a time?

Any questions you want to raise? Any suggestions you want to put forward?

*Thank you for your time and participation! We value all your comments and opinions. Together we will develop effective ways of improving the quality of antenatal care.*

# In-depth interview guide for Husband

**Perception of the husband of pregnant/postpartum women about Pregnancy care**

Do you think that a woman needs some extra care during her pregnancy? If you think so, what kinds of care are required during pregnancy?

What should a woman do or should not do during her pregnancy? [use hints: restriction to go outside, not to do work, food intake, etc.]

Who are the family members live with you? What kind of support they are providing now(ask separately for each adult family members even including young children as they sometimes help in HH chores)

How are decisions taken about her care? [About staying in natal /in-laws home, having food, antenatal care, etc.]

How can family members help her to attend the visit and taking care of her to follow the advice of Health care providers?

What complications/danger signs can occur during pregnancy? Source of information, compliance/non-compliance with the danger signs.

What will happen if pregnant women suffer from these danger signs? If pregnant women have any complications, what should the women and their families do? What can happen if they do not treat the complications?

Have you heard about anemia (pallor)/ hypertension (pressure)/ diabetes mellitus (sugar in the blood) like health problems during pregnancy?

How can a person identify these health problems during pregnancy?

If women suffer from anemia (pallor), hypertension (pressure), and Diabetes mellitus (sugar in the blood), what should do the pregnant women and her family members? What are the things (you and your baby) can happen if women do not treat these health problems? How can the complications be prevented for keeping pregnant women and her child healthy during pregnancy?

Do family members have any role in taking care of pregnant women during pregnancy with complications? Do health care providers have any role in taking care of women during pregnancy with complications?

**Awareness and perception regarding ANC, source of information, issues of ANC, Places to go for ANC**

What do you know about routine antenatal care visits during pregnancy? Do you think it is necessary at all for a woman during her pregnancy? If yes, why and when? If no, why not?

*If his wife did attend ANC visits:* Have your wife received any antenatal care visits? What benefit did she get from ANC?

*If his wife did not receive ANC visit:* Why did she not receive any ANC?

**ANC reminder and messages**

How can we effectively remind pregnant women about follow up ANC visits?

Will it help you if you receive a reminder for ANC visit through SMS to your mobile phone? What type of reminder would you like to get? What would be easier for you to understand the reminder and remember it? (Hints: SMS in Bengali or English, Voice call, voice SMS, etc.)

How can we aware of a pregnant woman and her family member if she found any risk factor during her ANC check-up so that she can visit qualified health providers at the right time? [Hints: SMS in Bengali or English, Voice call, voice SMS, etc.].

To whom should we contact for sending messages directly to pregnant women or her family members or both at a time?

Any questions you want to ask? Any suggestions you want to put forward?

*Thank you for your time and participation! We value all your comments and opinions. Together we will develop effective ways of improving the quality of antenatal care.*

# In-depth interview guide for Mothers-in-law

**Perception of the Mother in law of pregnant/postpartum women about Pregnancy care**

Do you think that a woman needs some extra care during her pregnancy? If you think so, what kinds of care are required during pregnancy?

What should a woman do or should not do during her pregnancy? [use hints: restriction to go outside, not to do work, food intake, etc.]

Who are the family members live with you? What kind of support they are providing now (ask separately for each adult family members even including young children as they sometimes help in HH chores)

How are decisions taken about her care? [About staying in natal /in-laws home, having food, antenatal care, etc.]

How can a family member help your daughter in law to attend the visit and to follow the advice of Health care provider?

What complications/danger signs can occur during pregnancy? Source of information, compliance/non-compliance with the danger signs.

What will happen if pregnant women suffer from these danger signs? If pregnant women have any complications, what should the women and their families do? What can happen if they do not treat the complications?

Have you heard about anemia (pallor)/ hypertension (pressure)/ diabetes mellitus (sugar in the blood) like health problems during pregnancy?

How can a person identify these health problems during pregnancy?

If women suffer from anemia (pallor), hypertension (pressure), and Diabetes mellitus (sugar in the blood), what should do the pregnant women and her family members? What are the things (you and your baby) can happen if women do not treat these health problems? How can the complications be prevented for keeping pregnant women and her child healthy during pregnancy?

Do family members have any role in taking care of pregnant women during pregnancy with complications? Do health care providers have any role in taking care of women during pregnancy with complications?

**Awareness and perception regarding ANC, source of information, issues of ANC, Places to go for ANC**

What do you know about routine antenatal care visits during pregnancy? Do you think it is necessary at all for a woman during her pregnancy? If yes, why and when? If no, why not?

*If her daughter in law did attend ANC visits:* Have your *daughter in law* received any antenatal care visits? What benefit did she get from ANC?

*If her daughter in law did not receive ANC visit:* Why did she not receive any ANC?

**ANC reminder and messages**

How can we effectively remind pregnant women about follow up ANC visits?

Will it help you if you receive a reminder for ANC visit through SMS to your mobile phone? What type of reminder would you like to get? What would be easier for you to understand the reminder and remember it? (Hints: SMS in Bengali or English, Voice call, voice SMS, etc.)

How can we aware of a pregnant woman and her family member if she found any risk factor during her ANC check-up so that she can visit qualified health providers at the right time? [Hints: SMS in Bengali or English, Voice call, voice SMS, etc.].

To whom should we contact for sending messages directly to the pregnant woman or her family members or both at a time?

Any questions you want to ask? Any suggestions you want to put forward?

*Thank you for your time and participation! We value all your comments and opinions. Together we will develop effective ways of improving the quality of antenatal care.*
