# Supplementary material for: Developing targeted client communication messages to pregnant women in Bangladesh: a qualitative study
Source: BMC Public Health. 2021 Apr 20;21:759. doi: 10.1186/s12889-021-10811-y (PMC8056650; doi:10.1186/s12889-021-10811-y)
Supplement: Supplementary file 2 — Additional file 2. Background information. [file 12889_2021_10811_MOESM2_ESM.docx]

# Background information of pregnant women

Study ID:

Name:

Age:

Parity:

Occupation:

Education:

Husband’s name:

Husband’s education:

Husband’s occupation:

Duration of pregnancy:

# Background information of postpartum women

Study ID:

Name:

Age:

Parity:

Education:

Occupation:

Husbands name:

Husband’s education:

Husband’s occupation:

Date of birth of the newborn:

# Background information of husband

Study ID:

Name:

Age:

Occupation:

Education:

Numbers of children:

# Background information of mother in law

Study ID:

Name:

Age:

Education:

Occupation:
